# Sanitary Shaving

**Published:** 1909-10

**Authors:** 


SANITARY SHAVING.—Now that men are shaving themselves more, you no doubt have frequent requests to prescribe an antiseptic that is at once effective and cheap. Tyree's Antiseptic Powder fills the bill. It has the strongest germicidal power—1 part in 50 of water kills every germ and bacteria—linked with extremely low cost. One ounce of the powder dissolved in a gallon of water makes an antiseptic solution that keeps the face smooth and healthy.

The human face was never intended to be scraped over by a razor. No matter how skillful a shaver may be the delicate cuticle of the face is lacerated and torn by the shaving process. An examination of the skin through a magnifying glass will prove this statement.

That is why men use some healing agent after shaving. The skin requires it. The better the healing agent the less discomfort to the shaver. Tyree's Antiseptic Powder is better than witch hazel, bay rum, and all other lotions commonly used for this purpose. It is also a great deal cheaper. Two gallons of antiseptic, made by dissolving two ounces of Tyree's Antiseptic Powder in two gallons of water, costs 25 cents—enough to last two months.

Try it after shaving yourself and you certainly will come to praise and prescribe it for face antisepsis.

---

THERE exist a number of cutaneous disorders which in the main are due to a general bad state of the tissues. It is in these that a general upbuilding process must be inaugurated in order to heal and improve the local cutaneous disturbance. It was formerly the custom to order cod liver oil, with good results. Today, it is equally advantageous to give the Cord. Ext. Ol. Morrhuae Comp. (Hagee), which acts not only as well but better, and is devoid of grease.—*American Journal of Dermatology.*

